# Supplementary material for: Fosl2 facilitates chromatin accessibility to determine developmental events during follicular maturation
Source: Nat Commun. 2025 Oct 8;16:8955. doi: 10.1038/s41467-025-64009-6 (PMC12508199; doi:10.1038/s41467-025-64009-6)
Supplement: Supplementary file 1 — Supplementary Information [file 41467_2025_64009_MOESM1_ESM.pdf]

Fosl2 facilitates chromatin accessibility to determine developmental events during follicular maturation

Zhang et al.

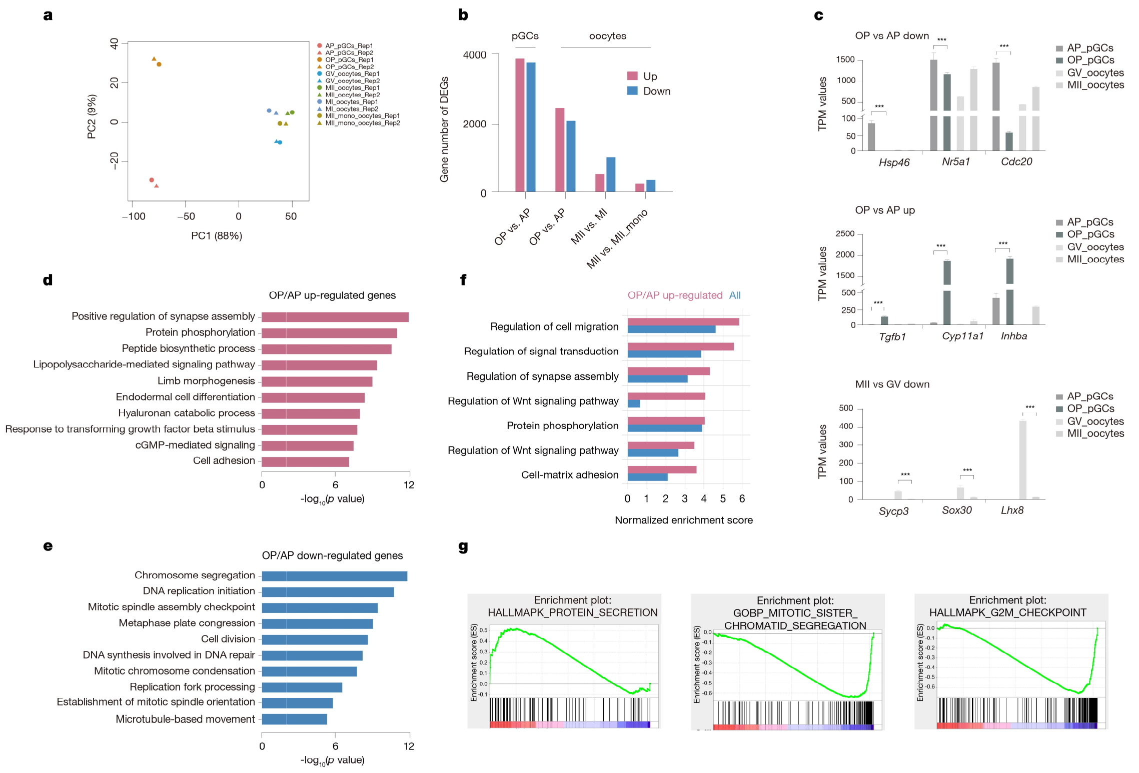

Supplementary Fig.1 | Dynamics of the transcriptional profiles during follicular maturation. **a**, Principal Component Analysis (PCA) displaying the correlation and dispersion of RNA-seq of pGCs and oocytes under different maturation phases. There are two independently performed biological replicates for each passage number (Rep 1 and 2). MII_mono, oocytes at the MII stage without pGCs support. **b**, Box plots showing the DEGs of pGCs and oocytes under different maturation phases. Differentially expressed genes (DEGs) are identified by R package DESeq2 with log2 (fold-change) ≥ 1 and adjusted p value < 0.05. **c**, Bar plots showing the representative gene expression (TPM) of RNA-seq under different maturation phases. Two biological replicates are independently performed for each sequencing. Error bars indicate the mean ±S.E.M. The p value was generated from a two-sided Student's t-test. $^{***}p < 0.001$. **d** and **e**, GO enrichments of the **d** up- and **e** down-regulated genes comparing the ovulatory phase with the antral phase. Bar length represents the enriched p value for biological processes. The p value was generated from a one-sided Hypergeometric test. **f,** Follicular maturation-related GO term enrichment scores within all and upregulated genes from the transcriptome dataset in pGCs. **g**, GSEA showing the enrichment of protein secretion, mitosis and G2M checkpoint pathway during follicular maturation.

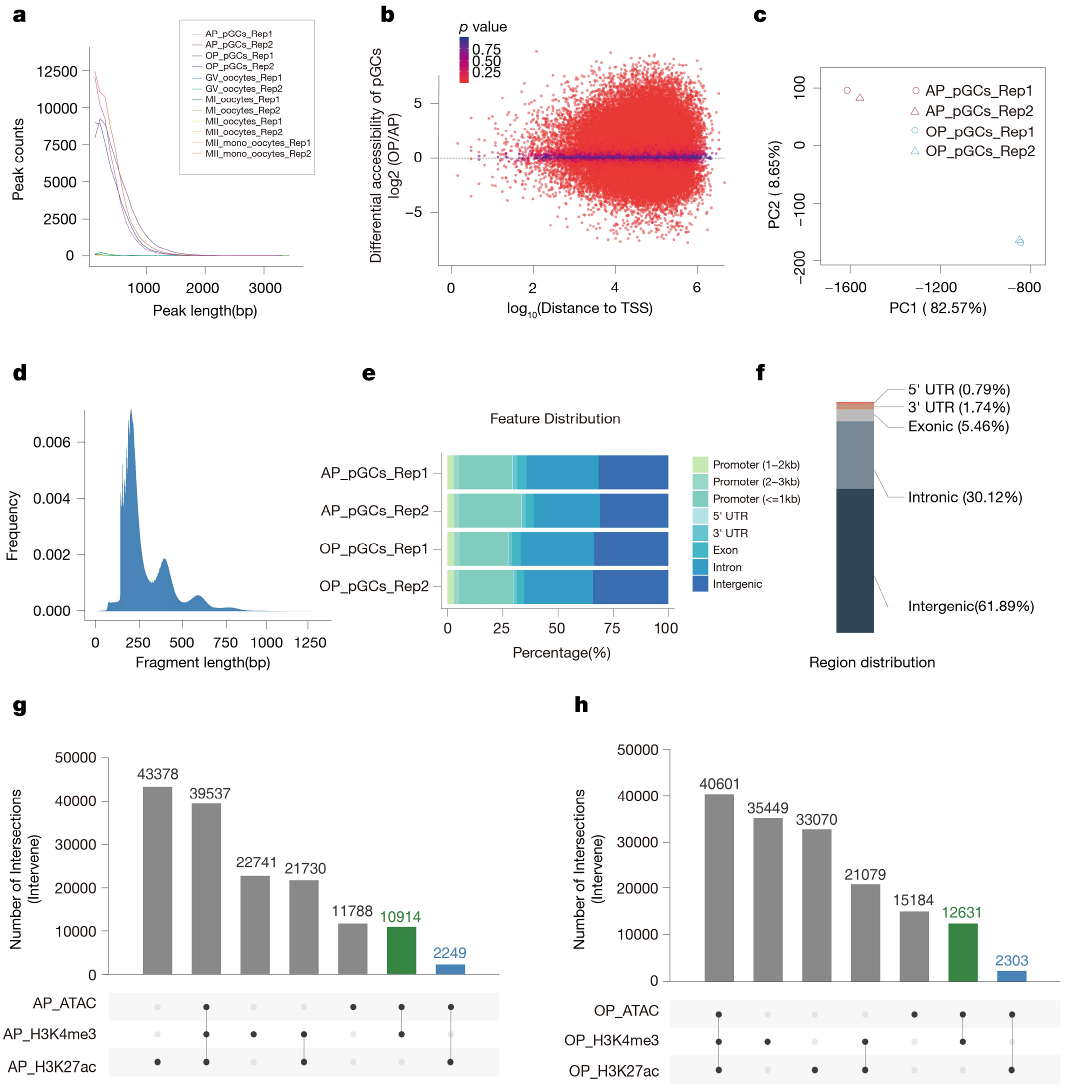

Supplementary Fig.2 | Dynamics of chromatin accessibility during follicular maturation. **a**, The distribution of the peak length with peak counts of accessibility regions under different maturation phases. **b**, Scatter plot of ATAC-seq signals in pGCs showing the accessibility change at the antral and ovulatory phases. The *p* value was generated from a two-sided Wald test. **c**, Principal Component Analysis (PCA) displaying the correlation and dispersion of ATAC-seq under different maturation phases. **d**, Representative distribution of insert size showing clear signal modulation for mono- and di-nucleosomes. **e**, Genomic feature distributions of accessible chromatin regions that changed in follicular maturation. UTR, untranslated region. **f**, Annotations of the genomic distribution of all peaks identified using ATAC-seq. **g** and **h,** Bar plots displaying the distribution of ATAC-seq signals at the antral and ovulatory phases in pGCs with H3K4me3 and H3K27ac CUT&Tag signals using the Intervene tool. Intersection numbers are labeled.

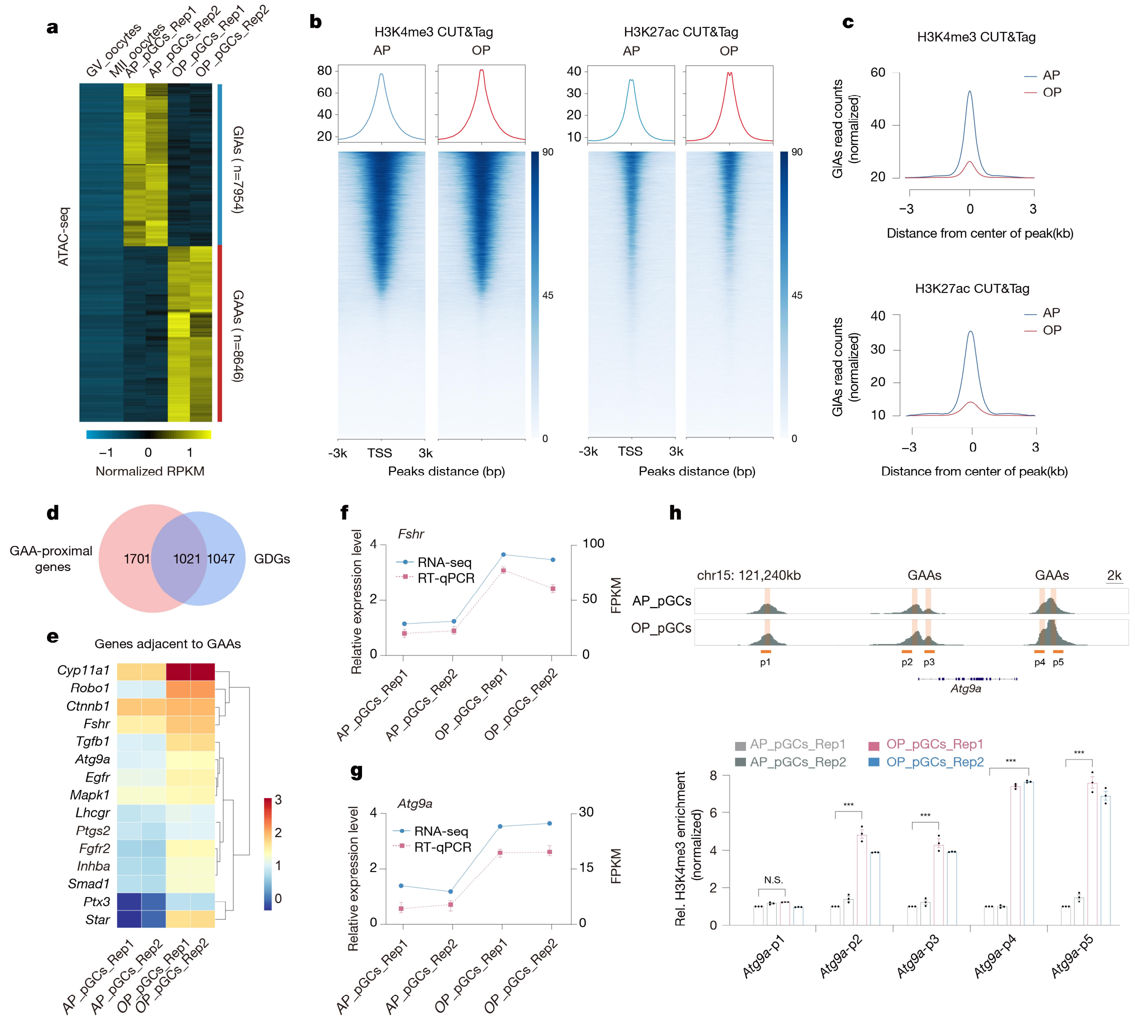

Supplementary Fig.3 | GAAs are responsible for robust adjacent GDG expression. a, Heatmap of ATAC-seq signals in pGCs showing the accessibility remodeling of GAAs and GIAs. Numbers of differential accessibility regions are labeled. **b**, Heatmaps and enrichment plots showing normalized read densities of H3K4me3 (left) and H3K27ac (right) CUT&Tag signals both at the antral and ovulatory phases. Tracks are centered at the peaks and extend ±3 kb. **c**, Enrichment plots displaying normalized GIA read densities for H3K4me3 and H3K27ac CUT&Tag signals. Tracks are centered at the TSS and extend ±3 kb. **d**, Venn diagram showing the overlap between GAA-proximal genes and identified GDGs list. **e**, Heatmap showing the expression of representative GDGs in proximity to GAAs (selected from the overlap genes in **d**). **f** and **g**, The expression of **f** *Fshr* and **g** *Atg9a* in pGCs during follicular maturation. The dashed line indicates RT-qPCR results, and the solid line indicates RNA-seq data. Error bars indicate the mean ±S.E.M. (n = 3 biological replicates). Source data are provided as a Source Data file. **h**, Location diagram of H3K4me3 ChIP-qPCR primers within the *Atg9a* locus (upper). ChIP-qPCR is used to measure the relative H3K4me3 levels for GAAs

within the corresponding *Atg9a* gene at the antral and ovulatory phases in pGCs (lower). IgG is used as the negative control. The enrichment is normalized to a 1:10 dilution of the input. Error bars indicate the mean ±S.E.M. (n = 3 biological replicates). The *p* value was generated from a two-sided Student's *t*-test. N.S., not significant, $^{***}p < 0.001$.

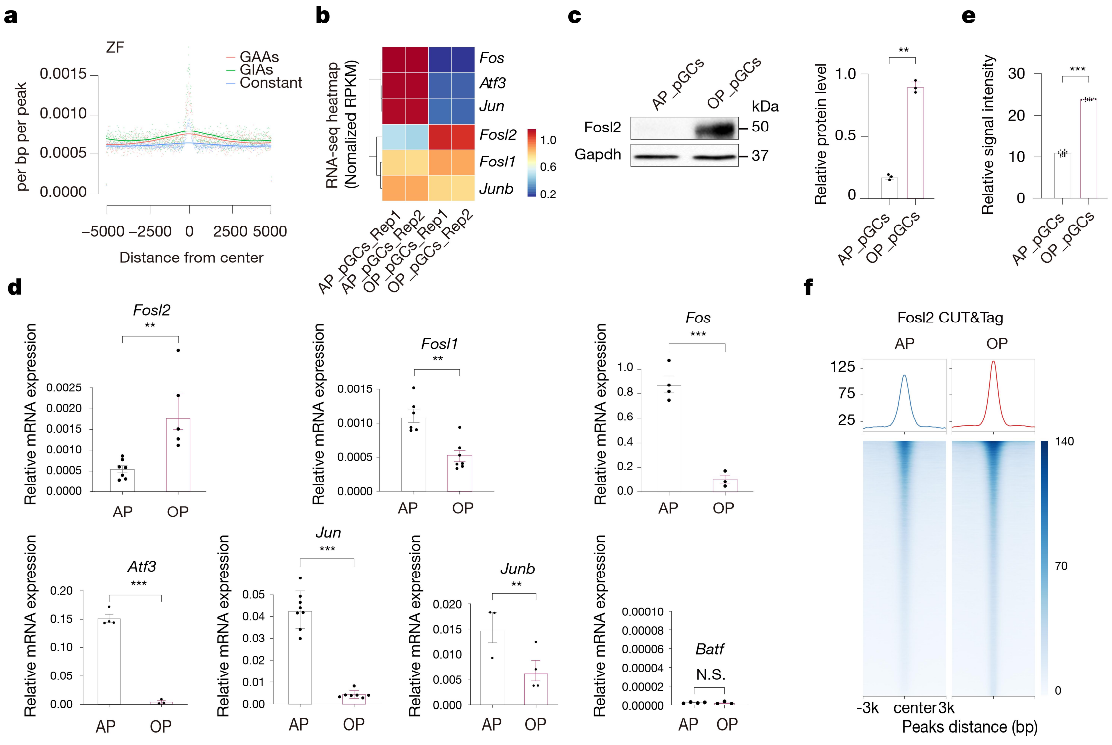

Supplementary Fig.4 | Fosl2 is enriched at GAAs. a, Position-weight matrices displaying the observed accessibility densities of GAAs, GIAs, and constant regions in ZF motif family. **b**, Heatmap showing the expression of AP-1 family genes of RNA-seq at the antral and ovulatory phases. **c**, Immunoblotting analysis (left) and quantification (right) of Fosl2 expression in pGCs under different maturation phases. Gapdh served as the loading control. Error bars indicate the mean ±S.E.M. (n = 3 biological replicates). The *p* value was generated from a two-sided Student's *t*-test. $^{**}p < 0.01$. Source data are provided as a Source Data file. **d**, The relative mRNA expression of *Fosl2* and six other genes in pGCs in the process of follicular maturation. Error bars indicate the mean ±S.E.M. (n = 3 biological replicates). The *p* value was generated from a two-sided Student's *t*-test. N.S., not significant. $^{**}p < 0.01$, $^{***}p < 0.001$. **e**, Immunofluorescence quantification of Fosl2 expression at the antral and ovulatory phases. Error bars indicate the mean ±S.E.M. (n = 3 biological replicates). The *p* value was generated from a two-sided Student's *t*-test. $^{***}p < 0.001$. **f**, Heatmaps and enrichment plots showing normalized read densities of Fosl2 CUT&Tag signals both at the antral and ovulatory phases. Tracks are centered at the peaks and extend ±3 kb.

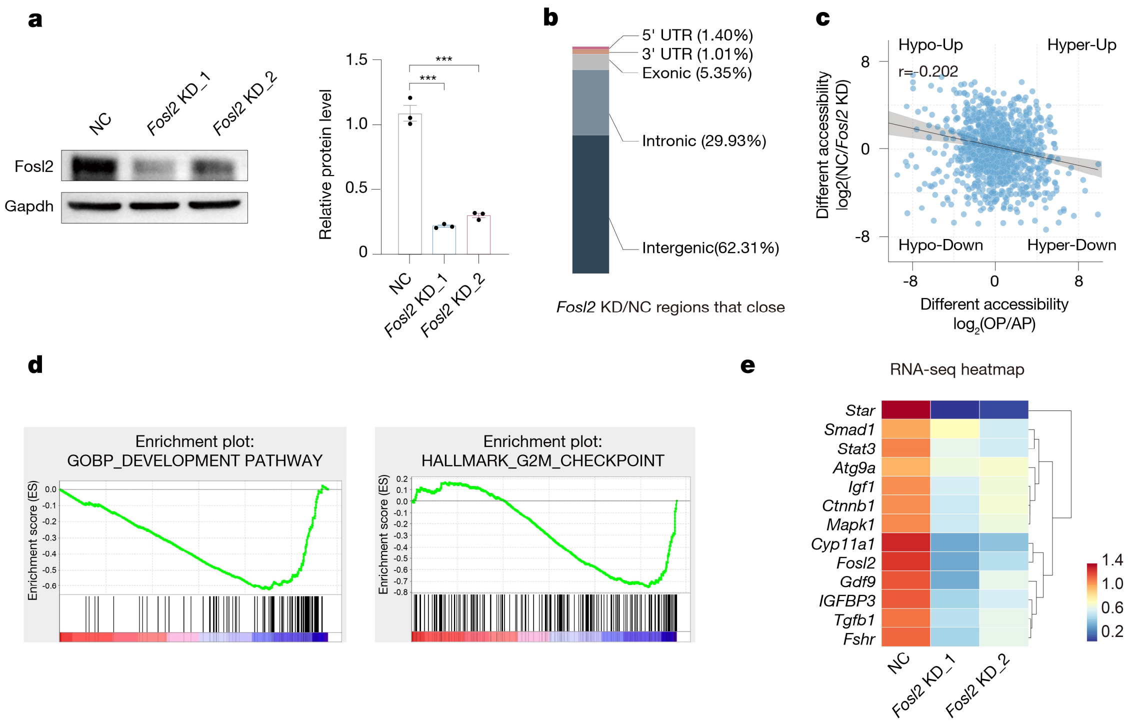

Supplementary Fig.5 | Fosl2 binds to GAAs to accelerate the transcription of GDGs. **a**, Immunoblotting analysis and quantification of Fosl2 after Fosl2 knockdown. Gapdh served as the loading control. Error bars indicate the mean ±S.E.M. (n = 3 biological replicates). The *p* value was generated from a two-sided Student's *t*-test. $^{***}p < 0.001$. Source data are provided as a Source Data file. **b**, Annotations of the genomic distribution of the peaks that close after Fosl2 silencing identified using ATAC-seq. **c**, Spearman's correlation of differential accessibility peaks between follicular maturation phases and accessibility regions that changed after Fosl2 knockdown (r = -0.202, *p* = 3.8e-6). The *p* value was generated from a two-sided test. **d**, GSEA showing the enrichment of developmental pathway and G2M checkpoint pathway following Fosl2 suppression. **e**, Heatmap displaying transcriptome change in expression of representative GDGs after Fosl2 knockdown.

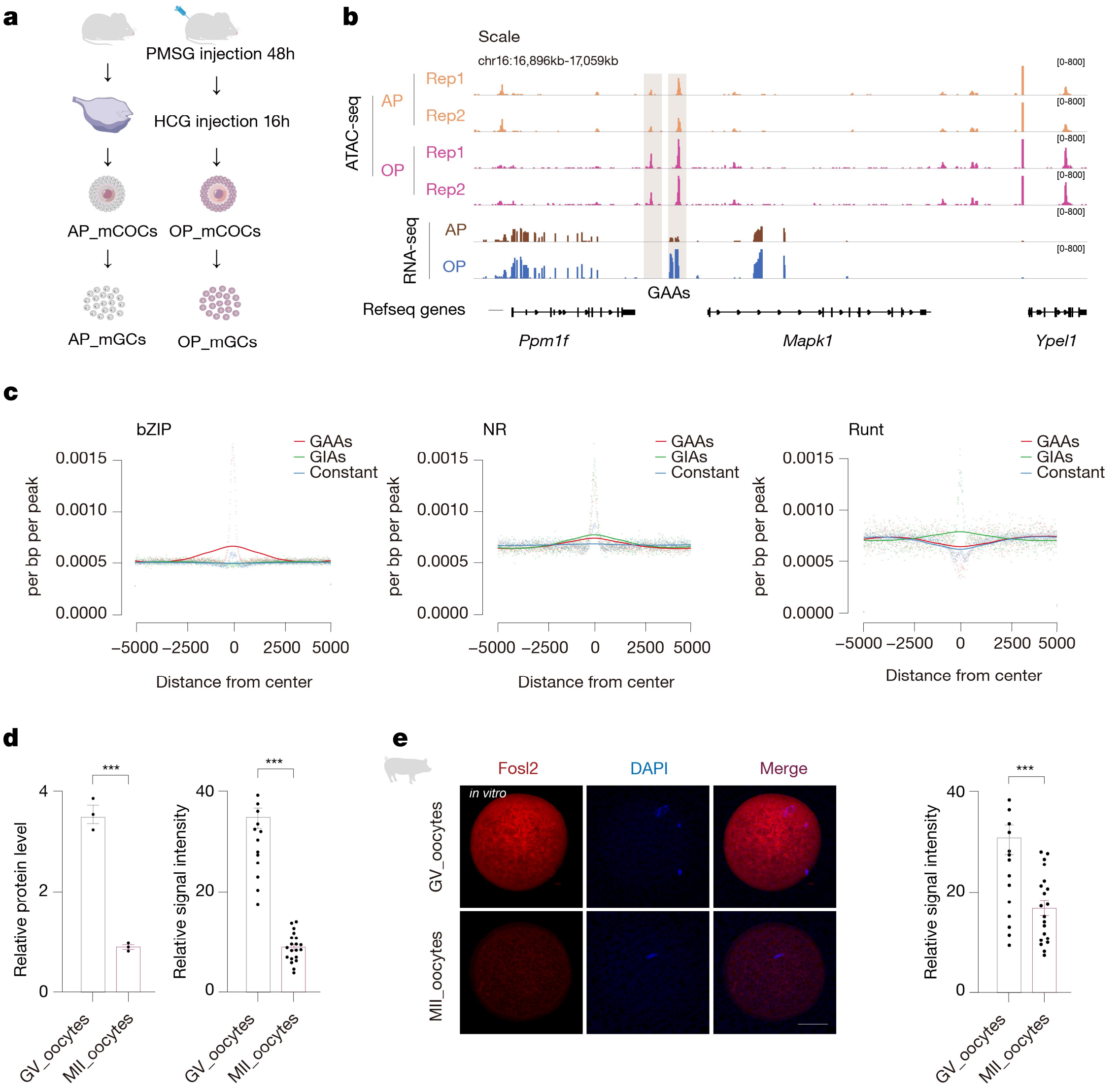

Supplementary Fig.6 | Fosl2-governed regulatory axis is conserved in mice. **a**, Schematic showing the acquisition of mGCs and oocytes under different maturation phases. PMSG, Pregnant Mare Serum Gonadotropin; HCG, human chorionic gonadotropin; mCOCs, murine cumulus-oocyte complexes. **b**, IGV snapshot displaying the ATAC-seq and RNA-seq signals during different maturation phases in mGCs at the representative gene *Mapk1* and *Ppm1f* loci. Vertical grey boxes indicate the potential GAAs predicted with ATAC-seq. **c,** Position-weight matrices displaying the observed accessibility densities of GAAs, GIAs, and constant regions in motif families defined in mGCs. **d**, Immunoblotting (left) and immunofluorescence (right) quantification of Fosl2 expression in murine oocytes at the GV and MII stages. Error bars indicate the mean ±S.E.M. (n = 3 biological replicates). The p value was generated from a two-sided Student's t-test. $^{***}p < 0.001$. Source data are provided as a Source Data file. **e**, Immunofluorescence staining and quantification of Fosl2 expression in porcine oocytes at the GV and MII stages. The position of the nucleolus is indicated

by DAPI staining. Results shown are representative of n = 3 biologically independent experiments with similar results. Scale bar, 25 µm. Error bars indicate the mean ±S.E.M. (n = 3 biological replicates). The *p* value was generated from a two-sided Student's *t*-test. $^{***}p < 0.001$.

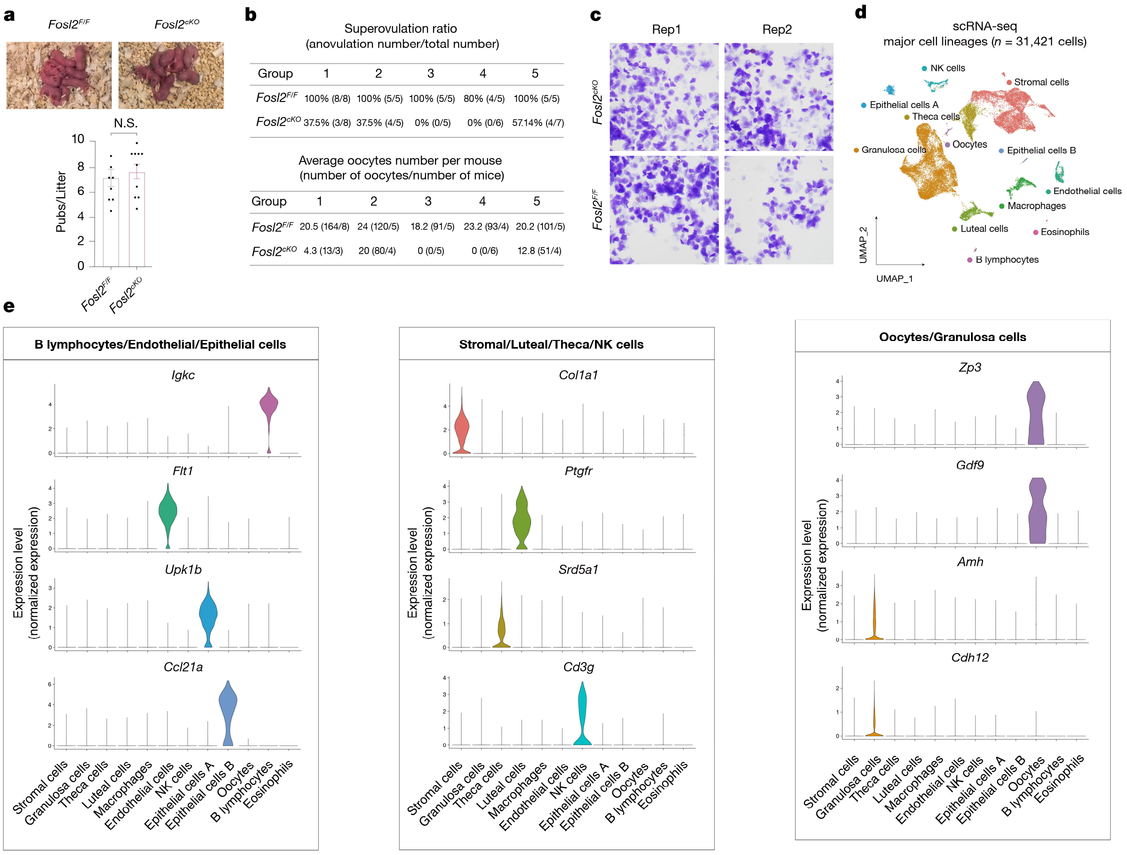

Supplementary Fig.7 | scRNA-seq of the mouse ovary after deletion Fosl2 in mGCs. a, Pubs per litter and quantification of 2-month-old *Fosl2*$^{F/F}$ and *Fosl2*^{cKO} female mice after mating with 2-month-old males. Error bars indicate the mean ±S.E.M. (*Fosl2*$^{F/F}$, n = 8 biological replicates, *Fosl2*^{cKO}, n = 10 biological replicates). The *p* value was generated from a two-sided Student's *t*-test. N.S., not significant. Source data are provided as a Source Data file. **b**, Tables showing the superovulation ratio and average oocytes number in five different groups of *Fosl2*$^{F/F}$ and *Fosl2*^{cKO} mice after superovulation. **c**, Vaginal cytology smear analyses showing the characteristic estrus-phase morphological features in *Fosl2*$^{F/F}$ and *Fosl2*^{cKO} mice that are subjected to scRNA-seq. Results shown are representative of n = 3 biologically independent experiments with similar results. Scale bar, 50 µm. **d**, UMAP plot featuring different cell clusters of ovaries in 2-month-old female mice. Clustering analysis revealing twelve distinct ovarian cell populations. **e**, Violin plots of specific marker genes for each ovarian cell clusters.

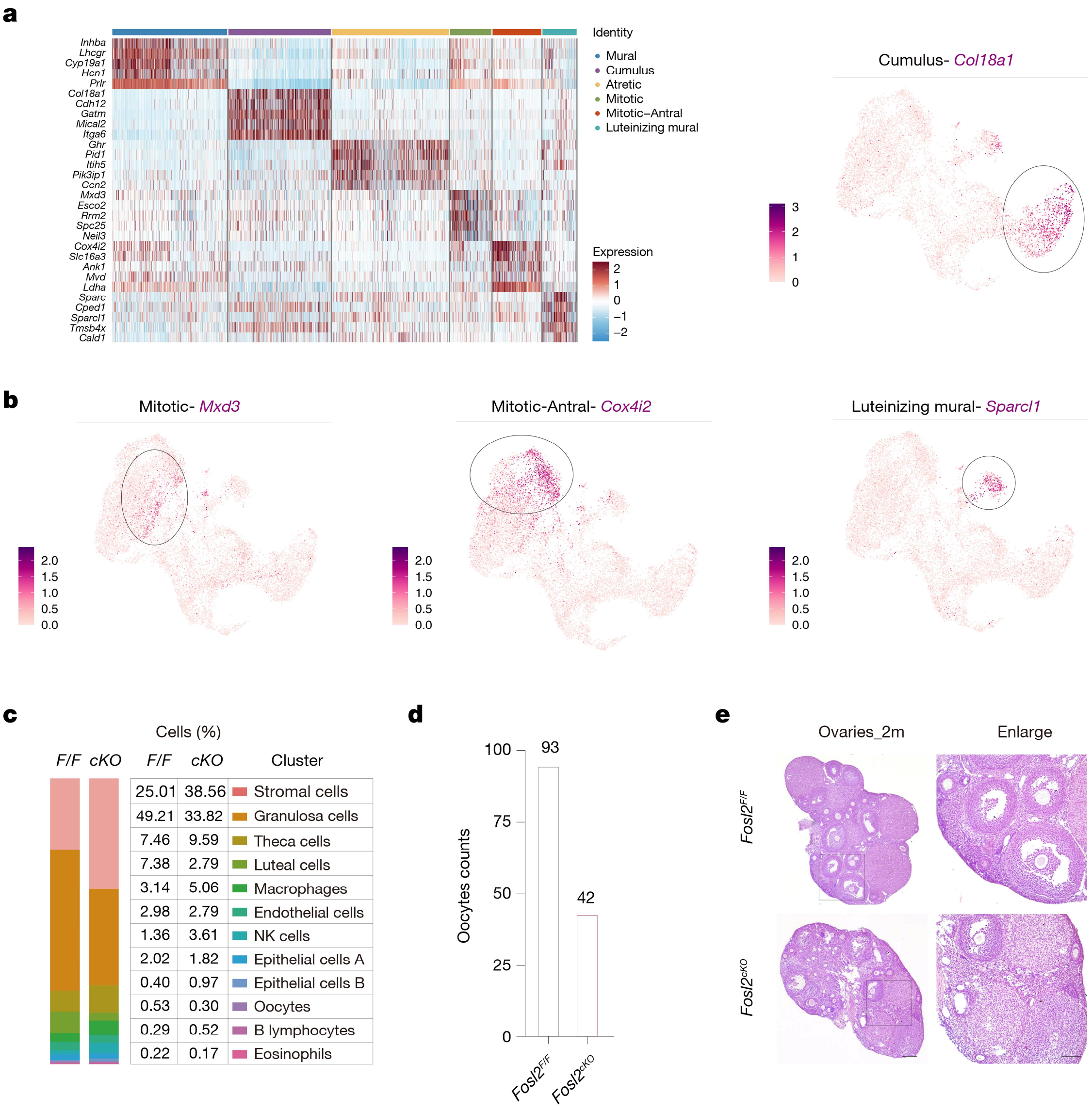

Supplementary Fig.8 | GC subclusters change after Fosl2 silencing. a, Heatmap of the top five markers of each GC subcluster by fold change. **b**, Gene marker identity of GC subclusters by UMAP plots. **c**, Numbers and percentages of major cell clusters in Fosl2$^{F/F}$ and Fosl2^{cKO} mice. **d**, Box plots showing the average oocyte counts in *Fosl2*$^{F/F}$ and *Fosl2*^{cKO} female mice. **e**, Histological images of ovaries of 2-month-old *Fosl2*$^{F/F}$ and *Fosl2*^{cKO} female mice. The enlarged views showing higher magnification of CL. Results shown are representative of n = 3 biologically independent experiments with similar results. Scale bar, left, 250 μm; right, 100 μm.
