# Supplementary material for: Fosl2 facilitates chromatin accessibility to determine developmental events during follicular maturation
Source: Nat Commun. 2025 Oct 8;16:8955. doi: 10.1038/s41467-025-64009-6 (PMC12508199; doi:10.1038/s41467-025-64009-6)
Supplement: Supplementary file 2 — Description of Additional Supplementary File [file 41467_2025_64009_MOESM2_ESM.pdf]

Description of Additional Supplementary Files

Supplementary Data 1: Primers used in experiments.

Supplementary Data 2: Differentially expressed genes in pCGs during follicular maturation.

Supplementary Data 3: List of GC-involved developmental genes (GDGs).

Supplementary Data 4: Summary of the sequencing data.

Supplementary Data 5: The locations and descriptions of GAA regions.

Supplementary Data 6: scRNA-seq makers in each ovary cluster.

Supplementary Data 7: scRNA-seq markers in each GC subcluster
