# Supplementary material for: Fosl2 facilitates chromatin accessibility to determine developmental events during follicular maturation
Source: Nat Commun. 2025 Oct 8;16:8955. doi: 10.1038/s41467-025-64009-6 (PMC12508199; doi:10.1038/s41467-025-64009-6)
Supplement: Supplementary file 10 — Reporting summary [file 41467_2025_64009_MOESM10_ESM.pdf]

nature portfolio

Corresponding author(s): Yiting Guan, Hongyong Zhang

Last updated by author(s): Aug 22, 2025

Reporting Summary

Nature Portfolio wishes to improve the reproducibility of the work that we publish. This form provides structure for consistency and transparency in reporting. For further information on Nature Portfolio policies, see our Editorial Policies and the Editorial Policy Checklist.

Statistics

For all statistical analyses, confirm that the following items are present in the figure legend, table legend, main text, or Methods section.

n/a	Confirmed	
☐	☒	The exact sample size (*n*) for each experimental group/condition, given as a discrete number and unit of measurement
☐	☒	A statement on whether measurements were taken from distinct samples or whether the same sample was measured repeatedly
☐	☒	The statistical test(s) used AND whether they are one- or two-sided<br>*Only common tests should be described solely by name; describe more complex techniques in the Methods section.*
☒	☐	A description of all covariates tested
☐	☒	A description of any assumptions or corrections, such as tests of normality and adjustment for multiple comparisons
☐	☒	A full description of the statistical parameters including central tendency (e.g. means) or other basic estimates (e.g. regression coefficient) AND variation (e.g. standard deviation) or associated estimates of uncertainty (e.g. confidence intervals)
☐	☒	For null hypothesis testing, the test statistic (e.g. *F*, *t*, *r*) with confidence intervals, effect sizes, degrees of freedom and *P* value noted<br>*Give P values as exact values whenever suitable.*
☒	☐	For Bayesian analysis, information on the choice of priors and Markov chain Monte Carlo settings
☐	☒	For hierarchical and complex designs, identification of the appropriate level for tests and full reporting of outcomes
☐	☒	Estimates of effect sizes (e.g. Cohen's *d*, Pearson's *r*), indicating how they were calculated

Our web collection on statistics for biologists contains articles on many of the points above.

Software and code

Policy information about availability of computer code

Data collection	ENCODE blacklist regions filtered out potential artifacts.
Data analysis	For bulk RNA-seq data processing, quality control was performed using FastQC, reads were aligned to mm10 or Sscrofa11.1 genomes using STAR (v2.7.3), and gene expression was quantified with HTseq-count. RUVseq (v1.34.0) was used to remove unwanted variation, and PCA was performed to assess data structure. Gene set enrichment analysis was conducted using fgse and visualized with clusterProfiler, using gene sets from MSigDB (https://www.gsea-msigdb.org/gsea/msigdb).<br>For ATAC-seq and CUT&Tag data processing, MACS2 called peaks with q < 0.05. Data quality metrics were calculated using deepTools (v3.5.1), and ChIPseeker (v1.44.0) annotated peak genomic features. Enriched motifs were identified using HOMER's findMotifsGenome.pl. Signal tracks were visualized in IGV (v2.14.1), and functional enrichment of genes near stage-specific distal peaks was analyzed using GREAT. Hierarchical clustering was performed using R's hclust() function.<br>For scRNA-seq data processing, raw sequencing data were processed using CellRanger (v3.1.0) against the GRCm38 mouse genome. Uniquely mapped reads were used for UMI counting and gene expression quantification. CellBender removed ambient RNA molecules, and cleaned matrices were analyzed with Seurat (v5.0.2). DoubletFinder identified and removed potential doublets. Data integration was performed using SCTransform with mitochondrial gene regression. Clustree package (v0.5.0) visualized hierarchical cluster relationships. Gene lists were analyzed in IPA_01.12 software.<br>No custom algorithm or software were generated in this study.

For manuscripts utilizing custom algorithms or software that are central to the research but not yet described in published literature, software must be made available to editors and reviewers. We strongly encourage code deposition in a community repository (e.g. GitHub). See the Nature Portfolio guidelines for submitting code & software for further information.

Data

Policy information about availability of data

All manuscripts must include a data availability statement. This statement should provide the following information, where applicable:

- Accession codes, unique identifiers, or web links for publicly available datasets
- A description of any restrictions on data availability
- For clinical datasets or third party data, please ensure that the statement adheres to our policy

Raw sequencing and processed data were deposited in the Gene Expression Omnibus (GEO) database, accessible using GSE267974. GEO accession codes for study data are RNA-seq, GSE267849; ATAC-seq, GSE267850; CUT&Tag, GSE267851; and scRNA-seq, GSE281100.

Research involving human participants, their data, or biological material

Policy information about studies with human participants or human data. See also policy information about sex, gender (identity/presentation), and sexual orientation and race, ethnicity and racism.

Reporting on sex and gender	N/A
Reporting on race, ethnicity, or other socially relevant groupings	N/A
Population characteristics	N/A
Recruitment	N/A
Ethics oversight	N/A

Note that full information on the approval of the study protocol must also be provided in the manuscript.

Field-specific reporting

Please select the one below that is the best fit for your research. If you are not sure, read the appropriate sections before making your selection.

☒ Life sciences ☐ Behavioural & social sciences ☐ Ecological, evolutionary & environmental sciences

For a reference copy of the document with all sections, see nature.com/documents/nr-reporting-summary-flat.pdf

Life sciences study design

All studies must disclose on these points even when the disclosure is negative.

Sample size	No statistical method was used to pre-determine the sample size. The sample sizes used in this study are indicated in the corresponding legends.
Data exclusions	Using the ENCODE blacklist regions for the mm10 genomic data allows for effective filtering out of potential technical artifacts.
Replication	Biological replicates of each experiments were described in the corresponding figure legends.
Randomization	No randomization was needed for data collection. Some presentation examples were randomly selected.
Blinding	The sequencing data in this study were processed and analyzed with computer programs. The investigators were not blinded during data collection and analysis. No blinding was required in this study.

Reporting for specific materials, systems and methods

We require information from authors about some types of materials, experimental systems and methods used in many studies. Here, indicate whether each material, system or method listed is relevant to your study. If you are not sure if a list item applies to your research, read the appropriate section before selecting a response.

Materials & experimental systems

n/a	Involved in the study
☐	☒ Antibodies
☐	☒ Eukaryotic cell lines
☒	☐ Palaeontology and archaeology
☐	☒ Animals and other organisms
☒	☐ Clinical data
☒	☐ Dual use research of concern
☒	☐ Plants

Methods

n/a	Involved in the study
☐	☒ ChIP-seq
☒	☐ Flow cytometry
☒	☐ MRI-based neuroimaging

Antibodies

Antibodies used

Fosl2, CST, 19967, 1:500;
Fshr, Proteintech, 22665, 1:500;
Mapk1, Proteintech,51068-1-AP, 1:500;
Fto, Proteintech, 27226-1-AP, 1:500;
Inhba, Bioss, bs-1774R, 1:500;
Ghr, Bioss, bs-0654R, 1:500;
Gapdh, Proteintech, 10494-1-AP, 1:1000;
β-actin, Proteintech, 20536-1-AP, 1:1000;
H3K4me3, Abcam, ab8580, 1:200;
H3K27ac, Abcam, ab4729, 1:200;
IgG, Proteintech, 30000-0-AP, 1:500.

Validation

The validation information can be found in the merchandise websites. Each antibody was titrated before use and the dilution ratio is shown below:
Fosl2 (CST, 19967) cited by 29 (https://www.cellsignal.cn/products/primary-antibodies/fra2-d2f1e-rabbit-mab/19967)
Fshr (Proteintech, 22665) cited by 3 (https://www.ptgcn.com/products/FSHR-Antibody-CL594-22665.htm#publications)
Mapk1 (Proteintech,51068-1-AP) cited by 42 (http://ptgcn.com/Products/ERK2-Antibody-51068-1-AP.htm#publications)
Fto (Proteintech, 27226-1-AP) cited by 179 (http://ptglab.co.jp/Products/FTO-Antibody-27226-1-AP.htm#publications)
Inhba (Bioss, bs-1774R) cited by 2 (http://www.bioss.com.cn/products/bs-1774R)
Ghr (Bioss, bs-0654R) cited by 15 (http://www.bioss.com.cn/products/bs-0654R)
Gapdh (Proteintech, 10494-1-AP) cited by 7678 (http://ptgcn.com/Products/GAPDH-Antibody-10494-1-AP.htm#publications)
β-actin (Proteintech, 20536-1-AP) cited by 4230 (https://www.ptglab.co.jp/products/ACTB-Antibody-20536-1-AP.htm#publications)
H3K4me3 (Abcam, ab8580) cited by 2132 (https://www.abcam.com/en-us/products/primary-antibodies/histone-h3-tri-methyl-k4-antibody-chip-grade-ab8580.html)
H3K27ac (Abcam, ab4729) cited by 2188 (https://www.abcam.cn/products/primary-antibodies/histone-h3-acetyl-k27-antibody-chip-grade-ab4729.html)
IgG (Proteintech, 30000-0-AP) cited by 525 (https://www.ptgcn.com/products/IgG-control-Antibody-30000-0-AP.htm#publications)

Eukaryotic cell lines

Policy information about cell lines and Sex and Gender in Research

Cell line source(s)

Porcine GCs (pGCs) and oocytes were isolated from fresh porcine COCs (pCOCs) obtained from slaughterhouse ovaries, and murine GCs (mGCs) and oocytes were isolated from fresh mCOCs obtained from the ovaries of 8-week-old female mice.

Authentication

Cell line were authenticated by morphology and genotyping.

Mycoplasma contamination

All cell lines were tested by mycoplasma contamination.

Commonly misidentified lines
(See ICLAC register)

None of the cell lines had commonly misidentified lines.

Animals and other research organisms

Policy information about studies involving animals; ARRIVE guidelines recommended for reporting animal research, and Sex and Gender in Research

Laboratory animals

Cyp19a1-Cre mice on a C57BL/6J genetic background (Nanjing, China). Floxed Fosl2 mice, Fosl2Flox/Flox (Fosl2F/F), were purchased from GemPharmatech Corporation (Jiangsu, China). Cyp19a1-Cre males were initially mated with Fosl2F/F females to generate males heterozygous for the Fosl2 floxed allele Cyp19a1-Cre; Fosl2+/Flox. Subsequently, these males were bred with Fosl2F/F females to produce female offspring with the Cyp19a1-Cre; Fosl2Flox/Flox (Fosl2cKO) genotype, and designated as Fosl2 conditional knockout mice.

Wild animals

Wild-type C57BL/6J mice were purchased from GemPharmatech Corporation (Jiangsu, China).

Reporting on sex	All mice involved in this experiment were female, except for the breeding males.
Field-collected samples	No field-collected samples were involved in this study.
Ethics oversight	This study was meticulously conducted in strict compliance with the ethical protocols approved by the Institutional Animal Care and Use Committee (IACUC) of Central People's Hospital of Zhanjiang, as authorized under Permission No. ZJDY2023-08.

Note that full information on the approval of the study protocol must also be provided in the manuscript.

Plants

Seed stocks	No plant samples were involved in this study.
Novel plant genotypes	No plant samples were involved in this study.
Authentication	No plant samples were involved in this study.

ChIP-seq

Data deposition

☒ Confirm that both raw and final processed data have been deposited in a public database such as GEO.

☒ Confirm that you have deposited or provided access to graph files (e.g. BED files) for the called peaks.

Data access links<br>*May remain private before publication.*	GEO accession codes for CUT&Tag is GSE267851.
Files in database submission	Processed uniquely mapped high quality reads in BW files were uploaded to GEO for downloading and downstream analysis.<br>GSM8279849 Cuttag_Sus_GCs_AP_IgG<br>GSM8279850 Cuttag_Sus_GCs_AP_Fosl2<br>GSM8279851 Cuttag_Sus_GCs_OP_IgG<br>GSM8279852 Cuttag_Sus_GCs_OP_Fosl2<br>GSM8279853 Cuttag_Sus_GCs_AP_H3K4me3<br>GSM8279854 Cuttag_Sus_GCs_AP_H3K27ac<br>GSM8279855 Cuttag_Sus_GCs_OP_H3K4me3<br>GSM8279856 Cuttag_Sus_GCs_OP_H3K27ac
Genome browser session<br>(e.g. UCSC)	All processed data can be visualized via the Integrative Genomics Viewer (IGV) web browser (https://igv.org/).

Methodology

Replicates	CUT&Tag in this study were performed with two biological replicates.
Sequencing depth	The selected DNA fragments were subjected to paired-end sequencing on the Novaseq 6000 platform to a depth of at least 2.5×107 reads.
Antibodies	H3K4me3, Abcam, ab8580;<br>H3K27ac, Abcam, ab4729;<br>Fosl2, CST, 19967;<br>IgG, Proteintech, 30000-0-AP.
Peak calling parameters	The bam file generated by the unique mapped reads was used as an input file, using MACS2 software for callpeak with cutoff q value < 0.05
Data quality	Data quality metrics for FRiP, NRF were calculated using deepTools (v3.5.1) and are detailed in Supplementary information.
Software	ENCODE blacklist regions filtered out potential artifacts, and MACS2 called peaks with q < 0.05. Data quality metrics were calculated using deepTools (v3.5.1), and ChIPseeker (v1.44.0) annotated peak genomic features. Enriched motifs were identified using HOMER's findMotifsGenome.pl. Signal tracks were visualized in IGV (v2.14.1), and functional enrichment of genes near stage-specific distal peaks was analyzed using GREAT. Hierarchical clustering was performed using R's hclust() function.
